# Grouping Gene Ontology terms to improve the assessment of gene set enrichment in microarray data

**DOI:** 10.1186/1471-2105-7-426

**Published:** 2006-10-03

**Authors:** Alex Lewin, Ian C Grieve

**Affiliations:** 1 Department of Epidemiology and Public Health, Imperial College, Norfolk Place, London W2 1PG, UK; 2 MRC Clinical Sciences Centre, Imperial College, Hammersmith Hospital, London W12 ONN, UK

## Abstract

**Background:**

Gene Ontology (GO) terms are often used to assess the results of microarray experiments. The most common way to do this is to perform Fisher's exact tests to find GO terms which are over-represented amongst the genes declared to be differentially expressed in the analysis of the microarray experiment. However, due to the high degree of dependence between GO terms, statistical testing is conservative, and interpretation is difficult.

**Results:**

We propose testing groups of GO terms rather than individual terms, to increase statistical power, reduce dependence between tests and improve the interpretation of results. We use the publicly available package POSOC to group the terms. Our method finds groups of GO terms significantly over-represented amongst differentially expressed genes which are not found by Fisher's tests on individual GO terms.

**Conclusion:**

Grouping Gene Ontology terms improves the interpretation of gene set enrichment for microarray data.

## Background

In recent years there has been an explosion in the number of studies measuring gene expression under various different experimental conditions. The outcome of such studies is usually a list of genes which have been seen to vary between the different conditions and therefore may be of interest to study further. Increasingly use is made of databases of other information on the genes in order to provide additional inference. One of the most used in the Gene Ontology (GO) database [1].

**Figure 1:**
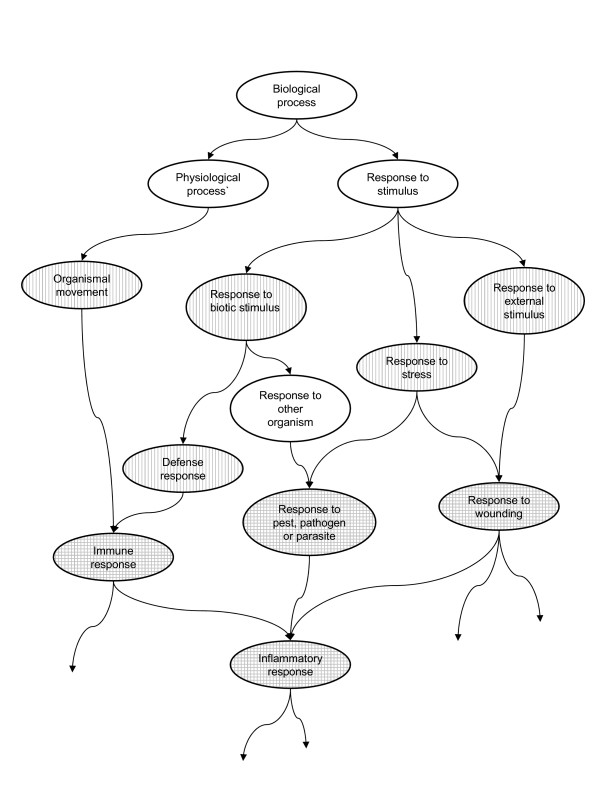
**A region of the GO graph**. Graph of all the ancestors of the term "inflammatory response" (terms shown in this figure may have child terms not shown). Vertical shading indicates terms with small p-values in FatiGO (though not significant). Horizontal shading indicates terms in the significant POSOC groups. Relations between GO terms were found using the QuickGO website: . The term "response to other organism" was not accessed by the FatiGO website.

The Gene Ontology is a database of standardised biological terms used to annotate gene products. In total it comprises around 16,000 terms, divided in three branches: Molecular Function, Biological Process and Cellular Component. Each branch can be represented as a directed acyclic graph (DAG) relating terms (or nodes) of different degrees of specificity, with directed links from less specific to more specific terms. Each node in the graph can have several parents (broader related terms) and children (more specific related terms). See Figure 1 for an example of a small section of the GO graph. Annotation of a gene to any given node A implies automatic annotation to all ancestors of A (the set of broader terms related to A by directed paths). Genes can be annotated with several terms, though many genes have not been annotated at all.

Tests for *gene set enrichment *[2] compare lists of differentially expressed (DE) genes and non-DE genes to find which terms in the GO are over or under-represented amongst the DE genes. Several groups have developed software to carry out Fisher's exact tests to find GO terms which are over-represented amongst the genes found to be differentially expressed in the microarray experiment, e.g. [3,4] and other work cited in [5]. The Fisher's test for node *i *essentially compares the proportion of DE genes annotated to node *i *with the proportion of non-DE genes annotated to node *i *(using the hypergeometric distribution).

Since there is a test for each of several 1000 GO nodes, multiple testing must be taken into account. This is generally done by controlling the False Discovery Rate (FDR) [6]. Benjamini and Hochberg's method [6] gives valid control of the FDR even when the different tests are positive-regression-dependent.

There remain some problems in the finding and interpretation of over or under-represented GO terms. Some difficulties which relate to the structure of the Gene Ontology graph are given here:

### I. Interpretation of closely related functions

Since all genes annotated to a given GO node are also annotated to all its parents, closely related nodes may be found separately significant. If the GO graph were a tree, this problem could be partly solved by choosing a depth of the tree to focus on, according to the balance of statistical power and specificity of function required. In fact most software packages available do treat the GO graph as if it were a tree, with levels defined as the shortest path from the node to the top. However, as the graph is not a tree, one level can contain child-parent pairs. (For example in Figure 1, the shortest paths from the root node "Biological Process" to "defense response" and "immune response" are of equal length, but "immune response" is a child of "defense response"). In the interpretation of the function of the DE genes, these should not really be treated separately.

### II. Statistical power

How far down the GO should we look? The most specific GO terms have few genes annotated so there is often not enough power to find these terms statistically significant. The more general the GO term, the more genes are annotated to it, but the less useful it is as an indication of the function of the differentially expressed genes.

### III. Dependence between tests

There is a great deal of positive dependence between GO terms, since many genes are annotated to several GO terms. This can be controlled for with an appropriate multiple testing correction, but with high dependence the correction will be very conservative, so some statistically significant terms will be missed.

Some work has been done to improve the dependence issue. Grossmann et al. 2006 [7] takes account of the hierarchical structure of the GO by measuring the over-representation of each term relative to its parent terms. Alexa et al. 2006 [8] downweights the contribution of genes to the calculation of over-representation of a term if the children of that term have already been found significantly enriched. These methods do not improve the statistical power, as the number of genes in each hypothesis test will be smaller than in the usual term-by-term tests, as double-counting is penalised. However, they do help with interpretation, since they produce just one (or at least not too many) significant p-values for each significant region of the graph. We propose a complementary approach; we choose to group closely related GO nodes together first, and obtain a p-value for each group. This has the additional benefit that we can find additional significant regions of the GO graph, as the power of the tests is improved by grouping terms together. We use a software package called the Poset Ontology Categorizer or POSOC [9] which groups GO terms together based on gene annotation and pseudo-distance between nodes, whilst respecting the structure of the Ontology. We call our method PoGO; software is available at .

## Results and discussion

### Wildtype versus knock-out mouse data set

Our first application is a data set consisting of wildtype and knock-out mice, referred to here as the Cd36 data set. Cd36 is a gene known to be involved in insulin-resistance [10]. This data set was previously analyzed with a Bayesian hierarchical model [11] and the resulting lists of genes input to the FatiGO software [3] to find over and under-represented GO terms. Controlling for the false discovery rate (using Benjamini and Hochberg's method [6]) led to no terms being found statistically significant. (The smallest Benjamini and Hochberg adjusted p-value is 0.27, so no significant results can be found at any sensible FDR level.)

**Figure 2:**
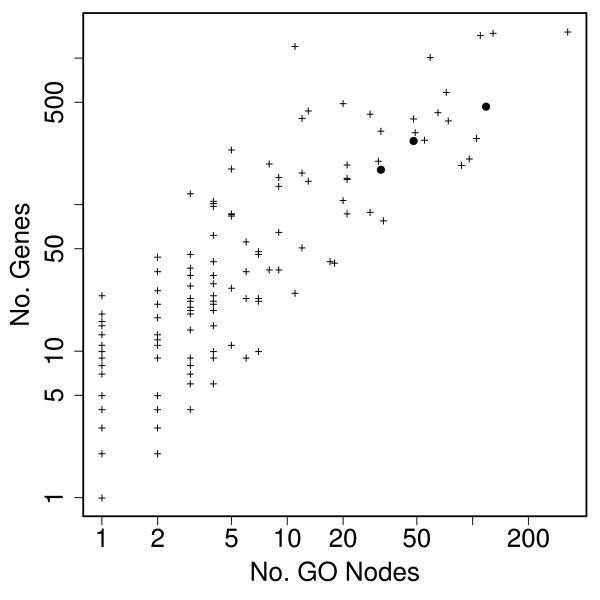
**Sizes of POSOC groups for the U74A chip**. Number of genes versus number of GO nodes for the POSOC groups for the U74A chip. The solid circles mark the three groups found significant in the group analysis for the Cd36 data set.

We now analyze this data using POSOC groups. This data set is from the U74A Affymetrix chip, so the POSOC groups we use are those found using all genes on the U74A chip (and the Biological Process branch of the GO). There are 258 groups. Note that the number of nodes in the Biological Process branch of the Gene Ontology is around 4100, so we have greatly reduced the space on which we perform the statistical tests. Table 1 shows the frequencies of group sizes. Figure 2 shows a scatter plot of the number of genes versus number of nodes. The distributions are highly skewed, with most groups being made up of fewer than 5 GO nodes, and having fewer than 50 genes annotated.

**Table 1:** Distribution of numbers of GO nodes in POSOC groups.

Size (no. GO nodes)	No. POSOC groups
1–5	190
6–10	28
11–15	6
16–20	6
21–40	12
41–60	2
61–80	4
81–100	5
101–120	1
121–140	1
> 140	3

Numbers of GO nodes in POSOC groups for the U74A chip (used for the Cd36 data set).

The adjusted p-values found by testing the POSOC groups are given in Table 2. PoGO finds three groups significantly over-represented amongst DE genes after controlling for FDR at 5%: those with cluster heads "response to pest, pathogen or parasite", "response to wounding" and "immune response". The numbers of GO nodes in these groups are 48, 32 and 118, and the numbers of genes 272, 173 and 467. The three groups are marked in Figure 2. It can be seen that they are large groups, but not atypical.

The GO categories found significant here are of interest. Previous research on the functions of Cd36 has linked the gene product to the regulation of immune responses to pathogens [12] and parasites [13]. Cd36 deficiency in humans has been found to be associated with increased severity of malarial pathology in affected populations [14].

**Table 2:** PoGO results for the Cd36 data set. Adjusted p-values for the 5 most significant POSOC groups.

POSOC group	Adjusted p-value
immune response	0.006
response to pest, pathogen or parasite	0.006
response to wounding	0.031
protein localization	0.100
digestion	0.522

### Chemically-induced diabetic rat data set

We have also applied PoGO to a publicly available list of differentially expressed genes found in an experiment on chemically-induced diabetic rats and normal non-induced rats, using Affymetrix arrays [15]. The original paper studied this in the context of erectile dysfunction in diabetes. The list of DE genes is given in the supplementary information for that paper.

The original work on this data set found interesting GO terms represented amongst the DE genes [15], but no multiple testing corrections were performed. We carried out a FatiGO analysis, and found 3 terms significantly over-represented for FDR controlled at 5%: "oxygen and reactive oxygen species metabolism", "response to oxidative stress" and "response to chemical stimulus", with adjusted p-values of 0.009, 0.003, and 0.03 respectively. The next smallest adjusted p-value is 0.11.

For this set of genes, 279 groups of GO terms are found by POSOC. Table 3 gives the most significant groups, with their adjusted p-values. PoGO finds 5 POSOC groups significant at FDR = 5%. The second group, "extracellular matrix organisation and biosynthesis" is of interest because it incorporates collagen and elastin related terms. This makes sense in the context of vascular changes in erectile dysfunction. The first and third, "lipid metabolism" and "oxygen and reactive oxygen species metabolism", are known to be relevant in diabetes (see for example [16] and [17]).

**Table 3:** PoGO results for Sullivan et al. data set. Adjusted p-values for the 10 most significant POSOC groups.

POSOC group	Adjusted p-value
lipid metabolism	0.012
extracellular matrix organisation and biosynthesis	0.012
oxygen and reactive oxygen species metabolism	0.027
copper ion homeostasis	0.030
acetyl-CoA metabolism	0.036
iron ion homeostatis	0.054
energy pathways	0.078
carboxylic acid metabolism	0.086
synaptic transmission	0.132
protein tetramerization	0.169

FatiGO finds terms corresponding to the third POSOC group, but not the first two.

### Transgenic and wildtype arabidopsis data set

Our third application of PoGO is to a data set from an experiment comparing transgenic and wildtype arabidopsis plants, using Affymetrix arrays [18]. The transgenic plants express a Brassica primary metabolic enzyme. They were observed in the original work to have enhanced levels of glucosinolate (GSL). The list of DE genes is given in the supplementary information for that paper.

PoGO finds three POSOC groups significant when controlling FDR at 5% (results are given in Table 4). The first one, "response to hormone stimulus", is a parent of a GO term found in [18]: "response to auxin stimulus". In the original work, patterns in expression profiles of known genes were identified that suggest perturbation of sulfate metabolism. The second and third POSOC groups found by PoGO, "sulfate assimilation" and "sulfate reduction, APS pathway", are close to "sulfate metabolism" in the GO graph. The next two terms, "response to temperature" and "cysteine metabolism", which have adjusted p-values of just over 5%, are also interesting. The former is a parent of the term "response to heat" identified in [18]. The term "cysteine metabolism" is also interesting in this context, as GSL is synthesized from methionine [19] and methionine is derived from cysteine [20]. This is also connected to "sulfate reduction, APS pathway", as the APS pathway in plants is part of the process of synthesizing cysteine from sulfate [21].

For this data set, FatiGO finds 10 significant GO terms (adjusted p-values are given in Table 5). FatiGO finds the terms "response to hormone stimulus", "response to temperature" and "sulfur utilization" (the last term is near to the other sulfur-related terms in the GO graph).

**Table 4:** PoGO results for Field et al. data set. Adjusted p-values for the 10 most significant POSOC groups.

POSOC group	Adjusted p-value
response to hormone stimulation	0.0001
sulfate reduction, APS pathway	0.0001
sulfate assimilation	0.032
response to temperature	0.055
cysteine metabolism	0.058
transcription, DNA-dependent	0.095
circadian rhythm	0.095
response to chitin	0.095
regulation of transcription	0.095
electron transport	0.123

**Table 5:** FatiGO results for Field et al. data set. Adjusted p-values for the 10 most significant GO terms.

POSOC group	Adjusted p-value
cellular macromolecule metabolism	0.007
protein metabolism	0.007
response to abiotic stimulus	0.009
cellular protein metabolism	0.010
response to hormone stimulation	0.041
response to temperature	0.041
sulfate utilization	0.041
response to endogenous stimulus	0.041
macromolecule stimulus	0.041

All other adjusted p-values are greater than 0.1

### Comparison of individual and group analyses

Here we present a comparison of the individual and group analyses for the Cd36 data set. Table 6 shows the GO terms ranked the highest by FatiGO (*i.e*. terms with the smallest p-values), with their membership of POSOC groups. Note that none of these terms were found significant in the FatiGO analysis. It can be seen that 10 out of the 15 top-ranked terms are members or immediate ancestors of at least one significant POSOC group. The others are too high up in the graph (i.e too general) to belong to POSOC groups. The most general GO terms are not very useful for functional analysis, as they are not specific enough. We see that grouping terms which are close in the graph has enabled us to find a significant region of the graph, where the individual term tests were not found significant.

**Table 6:** POSOC membership for top FatiGO terms for the Cd36 data set.

FatiGO Rank: GO node	Membership of POSOC groups	POSOC group significant at 5%?
1: response to external stimulus	IA	
2: response to pest, pathogen or parasite	response to pest, pathogen or parasite	yes
3: response to wounding	response to wounding	yes
4: organismal movement	IA	
5: response to biotic stimulus	IA	
6: neurophysiological process	-	
7: response to stress	IA	
8: inflammatory response	immune response	yes
	response to pest, pathogen or parasite	yes
	response to wounding	yes
9: transmission of nerve impulse	-	
10: neuromuscular physiological process	-	
11: defense response	IA	
12: immune response	immune response	yes
	response to pest, pathogen or parasite	yes
	response to wounding	yes
13: chemotaxis	immune response	yes
	response to pest, pathogen or parasite	yes
	cell migration	no
	chemotaxis	no
	response to wounding	yes
14: nucleobase, nucleoside, nucleotide and nucleic acid metabolism	-	
15: cell-cell signalling	-	

POSOC group membership of the most over-represented GO terms found by FatiGO. "IA" in the membership column indicates the term is an immediate ancestor (IA) of one of the significant POSOC groups. Note that none of these terms were found significant by the FatiGO analysis.

Not only has grouping terms improved the statistical power, it has also improved the interpretation of the results. Figure 1 shows part of the ontology containing several of the most over-represented GO terms from the FatiGO analysis (these terms have vertical shading). As shown in the graph, these are very close to each other in the ontology. Thus it makes sense to declare the region significantly over-represented rather than looking at each term separately.

Figure 1 includes the cluster heads of all three significant POSOC groups (terms in the significant POSOC groups have horizontal shading). The Figure shows that these groups overlap. Note that the Figure does not show all the descendant terms of the cluster heads. The "immune response" group includes 42 out of 48 of the "response to pest, pathogen or parasite" GO nodes and 28 out of 32 of the "response to wounding" GO nodes. The POSOC grouping reduces the number of tests we need to perform, but retains dependence between groups. The multiple testing correction used allows for this dependence, however.

One way of thinking about the amount of dependence between the statistical tests is to calculate the number of tests each gene appears in, since genes being annotated to multiple GO terms causes dependence between them. For the term-by-term analysis this is the number of individual GO terms a gene is annotated to. For the group analysis it is the number of POSOC groups the gene belongs to. Amongst the genes represented on the U74A chip, approximately 1/2 belong to fewer POSOC groups than individual terms, 3/8 belong to the same number of POSOC groups as individual terms, and 1/8 belong to more POSOC groups than individual terms (this is because some GO terms appear in more than one POSOC group). We see that there is an overall reduction in the number of tests each gene appears in, and therefore an overall reduction in the dependency between tests.

### Sensitivity to POSOC parameters

We have investigated the sensitivity of the PoGO results to changes in two POSOC parameters: firstly the choice of score function Sij(1)
 MathType@MTEF@5@5@+=feaafiart1ev1aaatCvAUfKttLearuWrP9MDH5MBPbIqV92AaeXatLxBI9gBaebbnrfifHhDYfgasaacH8akY=wiFfYdH8Gipec8Eeeu0xXdbba9frFj0=OqFfea0dXdd9vqai=hGuQ8kuc9pgc9s8qqaq=dirpe0xb9q8qiLsFr0=vr0=vr0dc8meaabaqaciaacaGaaeqabaqabeGadaaakeaacqWGtbWudaqhaaWcbaGaemyAaKMaemOAaOgabaGaeiikaGIaeGymaeJaeiykaKcaaaaa@3362@ or Sij(2)
 MathType@MTEF@5@5@+=feaafiart1ev1aaatCvAUfKttLearuWrP9MDH5MBPbIqV92AaeXatLxBI9gBaebbnrfifHhDYfgasaacH8akY=wiFfYdH8Gipec8Eeeu0xXdbba9frFj0=OqFfea0dXdd9vqai=hGuQ8kuc9pgc9s8qqaq=dirpe0xb9q8qiLsFr0=vr0=vr0dc8meaabaqaciaacaGaaeqabaqabeGadaaakeaacqWGtbWudaqhaaWcbaGaemyAaKMaemOAaOgabaGaeiikaGIaeGOmaiJaeiykaKcaaaaa@3364@ (see Methods Section for definitions), and secondly the choice of the specificity parameter *r*. We have performed this comparison on the Cd36 data set.

With the scoring function Sij(1)
 MathType@MTEF@5@5@+=feaafiart1ev1aaatCvAUfKttLearuWrP9MDH5MBPbIqV92AaeXatLxBI9gBaebbnrfifHhDYfgasaacH8akY=wiFfYdH8Gipec8Eeeu0xXdbba9frFj0=OqFfea0dXdd9vqai=hGuQ8kuc9pgc9s8qqaq=dirpe0xb9q8qiLsFr0=vr0=vr0dc8meaabaqaciaacaGaaeqabaqabeGadaaakeaacqWGtbWudaqhaaWcbaGaemyAaKMaemOAaOgabaGaeiikaGIaeGymaeJaeiykaKcaaaaa@3362@, changing specificity does not have a great effect. Using *r *= *log*_2_(2) POSOC obtains 249 cluster heads, which are quite general terms (we would not wish to decrease the specificity). Increasing *r *to *log*_2_(4) to increase the specificity of cluster heads in fact only gives 258 clusters (this is the set used in our main results above). Increasing *r *further does not give any appreciable increase in number of clusters. The list of most significant cluster heads found by PoGO using *r *= *log*_2_(2) is as in Table 2 except for the substitution of "cell activation" with an adjusted p-value of 0.12 in the fifth place. Using the second scoring function, *r *has a bigger effect. With *r *= *log*_2_(2) POSOC gives just 1 cluster, which is no use for our application. For *r *= *log*_2_(4) we find 221 clusters. The list of most significant cluster heads found by PoGO is again very similar to that found in the main analysis. "Response to pest, pathogen or parasite" and "response to wounding" are replaced by "response to external biotic stimulus" and "response to stress", which are very close in the GO graph. All that has changed is that slightly different terms have been designated as cluster heads.

## Conclusion

We have introduced the idea of finding regions of the GO graph which are over or under-represented amongst differentially expressed genes, rather than testing each GO node individually. We have applied the method to three data sets in three different organisms, one knock-out v. wildtype, one transgenic v. wildtype and one with a chemically induced phenotype. In all three cases, using the group analysis we find meaningful categories which would not have been found when testing individual GO terms.

The way in which GO terms are grouped together is crucial to the success of the group analysis. The POSOC method seems to be a sensible way to summarise the locations of the genes in the GO graph. It does however retain a considerable amount of dependence between the groups. This is inevitable, since annotation to any given GO term implies annotation to all ancestor terms (thus shared descendant terms of two terms in different groups must be assigned to both of those groups).

The cluster heads found by POSOC are rather general terms. From a biological point of view, it would of course be preferable to test more specific terms. We have found that in the current version of POSOC it is difficult to obtain more specific cluster heads. However, if we were to use groups of more specific terms we would start to run into the problems of increasing numbers of tests and few genes annotated to each group. We have found that with the current level of specificity, testing POSOC groups can find interesting results. In this implementation, we have not addressed the issues of uncertainties in gene annotation [22] or the uncertainty in which genes are differentially expressed: both the GO annotation IgiA
 MathType@MTEF@5@5@+=feaafiart1ev1aaatCvAUfKttLearuWrP9MDH5MBPbIqV92AaeXatLxBI9gBaebbnrfifHhDYfgasaacH8akY=wiFfYdH8Gipec8Eeeu0xXdbba9frFj0=OqFfea0dXdd9vqai=hGuQ8kuc9pgc9s8qqaq=dirpe0xb9q8qiLsFr0=vr0=vr0dc8meaabaqaciaacaGaaeqabaqabeGadaaakeaacqWGjbqsdaqhaaWcbaGaem4zaCMaemyAaKgabaGaemyqaeeaaaaa@31B1@ of gene *g *to node *i *and the differential expression status IgDE
 MathType@MTEF@5@5@+=feaafiart1ev1aaatCvAUfKttLearuWrP9MDH5MBPbIqV92AaeXatLxBI9gBaebbnrfifHhDYfgasaacH8akY=wiFfYdH8Gipec8Eeeu0xXdbba9frFj0=OqFfea0dXdd9vqai=hGuQ8kuc9pgc9s8qqaq=dirpe0xb9q8qiLsFr0=vr0=vr0dc8meaabaqaciaacaGaaeqabaqabeGadaaakeaacqWGjbqsdaqhaaWcbaGaem4zaCgabaGaemiraqKaemyraueaaaaa@316F@ of each gene are treated as fixed. The method could be extended to incorporate both types of uncertainty. Using a Bayesian model to analyse the gene expression data gives the whole posterior distribution of IgDE
 MathType@MTEF@5@5@+=feaafiart1ev1aaatCvAUfKttLearuWrP9MDH5MBPbIqV92AaeXatLxBI9gBaebbnrfifHhDYfgasaacH8akY=wiFfYdH8Gipec8Eeeu0xXdbba9frFj0=OqFfea0dXdd9vqai=hGuQ8kuc9pgc9s8qqaq=dirpe0xb9q8qiLsFr0=vr0=vr0dc8meaabaqaciaacaGaaeqabaqabeGadaaakeaacqWGjbqsdaqhaaWcbaGaem4zaCgabaGaemiraqKaemyraueaaaaa@316F@ for each gene. This uncertainty could be propagated to provide uncertainty on the Fisher p-values, or used as part of a unified Bayesian model resulting in posterior probabilities for GO groups being over or under-represented. This has been done by [23], in the context of testing individual GO terms. If the annotation uncertainty (uncertainty on IgiA
 MathType@MTEF@5@5@+=feaafiart1ev1aaatCvAUfKttLearuWrP9MDH5MBPbIqV92AaeXatLxBI9gBaebbnrfifHhDYfgasaacH8akY=wiFfYdH8Gipec8Eeeu0xXdbba9frFj0=OqFfea0dXdd9vqai=hGuQ8kuc9pgc9s8qqaq=dirpe0xb9q8qiLsFr0=vr0=vr0dc8meaabaqaciaacaGaaeqabaqabeGadaaakeaacqWGjbqsdaqhaaWcbaGaem4zaCMaemyAaKgabaGaemyqaeeaaaaa@31B1@) could be expressed in the form of a statistical distribution, it could be incorporated in a similar way, possibly in the form of a prior distribution in a Bayesian model.

## Methods

### Grouping nodes in the Gene Ontology

In order to overcome issue I given in the Introduction, we would like to group together GO terms which are close in the ontology. One way to do this would be to find the most significant individual GO terms and then group them for interpretation. This was done for example in [11]. Here we use software which groups the GO terms before statistical testing.

The Poset Ontology Categorizer (POSOC) [9] takes a list of genes and produces a list of GO nodes (called cluster heads) which summarize the distribution of gene annotations on the graph. It is based on the concepts of coverage (number of genes annotated to the group of GO terms) and specificity (how general the cluster heads are). A pseudo-distance is defined on the graph, which is the minimum path length between comparable nodes. Each node in the graph is scored by summing the inverse of the pseudo-distances to its descendant nodes where the genes are annotated (so a higher score indicates smaller distances and more genes covered). The nodes with the highest scores are denoted "cluster heads" and are used to summarize the gene annotation distribution.

There are two scoring functions available: Sij(1)
 MathType@MTEF@5@5@+=feaafiart1ev1aaatCvAUfKttLearuWrP9MDH5MBPbIqV92AaeXatLxBI9gBaebbnrfifHhDYfgasaacH8akY=wiFfYdH8Gipec8Eeeu0xXdbba9frFj0=OqFfea0dXdd9vqai=hGuQ8kuc9pgc9s8qqaq=dirpe0xb9q8qiLsFr0=vr0=vr0dc8meaabaqaciaacaGaaeqabaqabeGadaaakeaacqWGtbWudaqhaaWcbaGaemyAaKMaemOAaOgabaGaeiikaGIaeGymaeJaeiykaKcaaaaa@3362@ = (*δ*(*i*, *j*)^*r *^+ 1)^-1 ^and Sij(2)
 MathType@MTEF@5@5@+=feaafiart1ev1aaatCvAUfKttLearuWrP9MDH5MBPbIqV92AaeXatLxBI9gBaebbnrfifHhDYfgasaacH8akY=wiFfYdH8Gipec8Eeeu0xXdbba9frFj0=OqFfea0dXdd9vqai=hGuQ8kuc9pgc9s8qqaq=dirpe0xb9q8qiLsFr0=vr0=vr0dc8meaabaqaciaacaGaaeqabaqabeGadaaakeaacqWGtbWudaqhaaWcbaGaemyAaKMaemOAaOgabaGaeiikaGIaeGOmaiJaeiykaKcaaaaa@3364@ = (1 - *δ*(*i*, *j*))^*r*^, where *δ*(*i,j*) is the pseudo-distance between nodes *i *and *j *and the parameter *r *weights the distances and thus controls the specificity of the cluster heads. The actual score for node *i *is then ∑_*g *_∑_*j *_Sij(k)
 MathType@MTEF@5@5@+=feaafiart1ev1aaatCvAUfKttLearuWrP9MDH5MBPbIqV92AaeXatLxBI9gBaebbnrfifHhDYfgasaacH8akY=wiFfYdH8Gipec8Eeeu0xXdbba9frFj0=OqFfea0dXdd9vqai=hGuQ8kuc9pgc9s8qqaq=dirpe0xb9q8qiLsFr0=vr0=vr0dc8meaabaqaciaacaGaaeqabaqabeGadaaakeaacqWGtbWudaqhaaWcbaGaemyAaKMaemOAaOgabaGaeiikaGIaem4AaSMaeiykaKcaaaaa@33D1@/∑_*g *_*N*_*g*_, *k *= 1 or 2, where *N*_*g *_is the total number of annotations of gene *g *and the sum over *j *is the sum over all descendant nodes of *i *which annotate *g*. For the main analyses in this paper we use the first scoring function, with *r *= *log*_2_(4). We also look at the sensitivity to these choices in the Results section.

Each cluster head represents a group of GO nodes (all those comparable and more specific than the cluster head). We compare the proportion of differentially expressed and non-differentially expressed genes in each of these groups. We anticipate that with the grouping of terms issues II and III (statistical power and dependence) might be lessened.

### Statistical testing of groups of GO nodes

PoGO performs a Fisher's test on each GO group *i *= 1, ..., *m*. This tests for association between differential expression and GO annotation for the given group. Genes which have no annotations at all are not counted here (as in the usual Fisher's tests on individual GO terms). Gene annotations come from the NetAffx website [24]. Effectively, the input to each test is a pair of indicator functions for each gene, one indicating whether or not the gene is differentially expressed IgDE
 MathType@MTEF@5@5@+=feaafiart1ev1aaatCvAUfKttLearuWrP9MDH5MBPbIqV92AaeXatLxBI9gBaebbnrfifHhDYfgasaacH8akY=wiFfYdH8Gipec8Eeeu0xXdbba9frFj0=OqFfea0dXdd9vqai=hGuQ8kuc9pgc9s8qqaq=dirpe0xb9q8qiLsFr0=vr0=vr0dc8meaabaqaciaacaGaaeqabaqabeGadaaakeaacqWGjbqsdaqhaaWcbaGaem4zaCgabaGaemiraqKaemyraueaaaaa@316F@ and one indicating if the gene is annotated to the GO group *i *being tested IgiA
 MathType@MTEF@5@5@+=feaafiart1ev1aaatCvAUfKttLearuWrP9MDH5MBPbIqV92AaeXatLxBI9gBaebbnrfifHhDYfgasaacH8akY=wiFfYdH8Gipec8Eeeu0xXdbba9frFj0=OqFfea0dXdd9vqai=hGuQ8kuc9pgc9s8qqaq=dirpe0xb9q8qiLsFr0=vr0=vr0dc8meaabaqaciaacaGaaeqabaqabeGadaaakeaacqWGjbqsdaqhaaWcbaGaem4zaCMaemyAaKgabaGaemyqaeeaaaaa@31B1@. Thus the condition of being differentially expressed or not is considered known, as are the gene annotations. The result is a list of p-values, *p*_*i*_, *i *= 1, ..., *m*.

A decision rule is then found (a critical p-value below which the GO group is declared significantly over or under-represented in the DE genes) by controlling for the False Discovery Rate (FDR). We use the Benjamini and Hochberg method [6], which is valid under positive regression dependence [25]. Two variables are positive-regression-dependent if large values of one are associated with large values of the other. It implies, but is not implied by, positive correlation. It is not clear if the GO satisfies this condition, however we would expect a lot of positive dependency between GO terms, as the dependency is caused by the same genes being annotated to different terms. There are methods to control FDR under arbitrary dependence [25], but these are very conservative compared to the Benjamini and Hochberg method (and the Benjamini and Hochberg method is itself conservative as it includes no estimate of the number of true null hypotheses but takes this to be 1 in the calculation of FDR). Therefore we consider it acceptable to use the Benjamini and Hochberg method, which takes the risk of some false positives, whilst gaining in the number of true positives.

## Authors' contributions

AL conceived of the method, performed statistical analysis and drafted the manuscript. IG developed the software for the analysis, performed statistical analysis and investigated the biological interpretations of the results.
